# Transvaginal Appendectomy: A Systematic Review

**DOI:** 10.1155/2014/384706

**Published:** 2014-12-29

**Authors:** Mehmet Ali Yagci, Cuneyt Kayaalp

**Affiliations:** Department of Surgery, Turgut Ozal Medical Center, Inonu University, 44315 Malatya, Turkey

## Abstract

*Background*. Natural orifice transluminal endoscopic surgery (NOTES) is a new approach that allows minimal invasive surgery through the mouth, anus, or vagina.* Objective*. To summarize the recent clinical appraisal, feasibility, complications, and limitations of transvaginal appendectomy for humans and outline the techniques.* Data Sources*. PubMed/MEDLINE, Cochrane, Google-Scholar, EBSCO, clinicaltrials.gov and congress abstracts, were searched.* Study Selection*. All related reports were included, irrespective of age, region, race, obesity, comorbidities or history of previous surgery. No restrictions were made in terms of language, country or journal.* Main Outcome Measures*. Patient selection criteria, surgical techniques, and results.* Results*. There were total 112 transvaginal appendectomies. All the selected patients had uncomplicated appendicitis and there were no morbidly obese patients. There was no standard surgical technique for transvaginal appendectomy. Mean operating time was 53.3 minutes (25–130 minutes). Conversion and complication rates were 3.6% and 8.2%, respectively. Mean length of hospital stay was 1.9 days.* Limitations.* There are a limited number of comparative studies and an absence of randomized studies.* Conclusions*. For now, nonmorbidly obese females with noncomplicated appendicitis can be a candidate for transvaginal appendectomy. It may decrease postoperative pain and enable the return to normal life and work off time. More comparative studies including subgroups are necessary.

## 1. Introduction

Since its original description by Mc Burney in 1894, appendectomy has been one of the most common surgical procedures performed by surgeons. In the last decades, laparoscopic appendectomy was an increasingly accepted treatment method for acute appendicitis, particularly for obese or female patients [1]. Natural orifice transluminal endoscopic surgery (NOTES) is a new approach that allows for minimal invasive surgery through the natural orifices such as mouth, anus, or vagina. This technique aims to avoid any visible scars on the body surface. Less incision on the abdomen helps to reduce surgical pain, analgesic requirement, recovery time, hernia formation, intra-abdominal adhesion, and surgical site infection. However, NOTES has several disadvantages and limitations with the currently available instruments, including limited access and less familiar working angles and operative views. In the past few years, many centers have published their experiences with NOTES appendectomies in humans. This study aimed to summarize the recent clinical appraisals, feasibility, complications, and limitations of transvaginal appendectomy for humans and to outline techniques.

## 2. Material and Methods

Electronic searches in December 2013 of the PubMed/MEDLINE, Cochrane, Google Scholar, and EBSCOhost-Academic Search Complete, including CINAHL, used the key words [(vaginal OR transvaginal) AND (appendectomy OR appendectomies OR appendicectomy OR appendicectomies)]. All the studies including congress proceedings and abstracts that describe the clinical course of patients were accepted for the analysis. Two reviewers (Mehmet Ali Yagci and Cuneyt Kayaalp) assessed the list of titles and/or abstracts of the scanned articles at PubMed/MEDLINE and Cochrane using the key words in a function of [all fields]. If the articles met our inclusion criteria, full-text versions were obtained for assessment. If the articles were obviously irrelevant to the aim of this systematic review, they were excluded. Additional studies were also excluded due to their content (editorial letters, reviews, experimental studies, duplicated studies, technical notes not including patient data, and survey studies including questionnaires). After PubMed and Cochrane searches, we scanned the EBSCOhost-Academic Search Complete and Google Scholar databases with the same key words but using the [title] function. If any additional studies were found, they were added to the first search results. The unpublished, potentially relevant, trials at the registered trials database at https://www.clinicaltrials.gov/ were searched as well. The references of the selected relevant articles were cross checked to decrease the possibility of missing publications.

Transvaginal appendectomy was defined as a way of natural orifice translumenal endoscopic surgery for the appendix. Studies describing concomitant appendectomies during transvaginal hysterectomy were not considered for this systematic review. All the patients were included, irrespective of age, region, race, obesity, comorbidities, or history of previous surgery. No restrictions were made on language, country, or journal. In cases of disagreement during the study selection and analysis, the reviewers discussed the disagreement and a consensus was reached. Data for affiliation, number of the patients, age, clinical findings, inclusion and exclusion criteria, body mass index, history of previous abdominal surgery, trocar sites (pure or hybrid) and types, scope site and types (flexible or rigid), vaginal access and colpotomy closure techniques, intraoperative and postoperative complications, operating time, conversion to conventional laparoscopy or open surgery, postoperative pain, length of hospital stay, time off work, long term sexual function, and cosmetic satisfaction were recorded. A computer program including spreadsheet was used for records (Excel 2013, Microsoft Windows). If there were any missing data, we tried to contact the authors via e-mail.

Data were tabulated in tables, and column sums were created with the numbers or the means ± standard deviations, or the ranges of relevant parameters. When studies reported the median and range, we estimated the mean and standard deviation using the method described by Hozo et al. [2]. Basic calculations were used for the total numbers of the dichotomous outcomes and weighted means with ranges for the continuous outcomes. Chi-square test or the Fisher exact test (if expected values were less than 5) and Student's *t*-test were used for statistical analysis of both dichotomous and continuous variables (SPSS 13.0). A *P* value <.05 was considered significant.

## 3. Results

**Figure 1:**
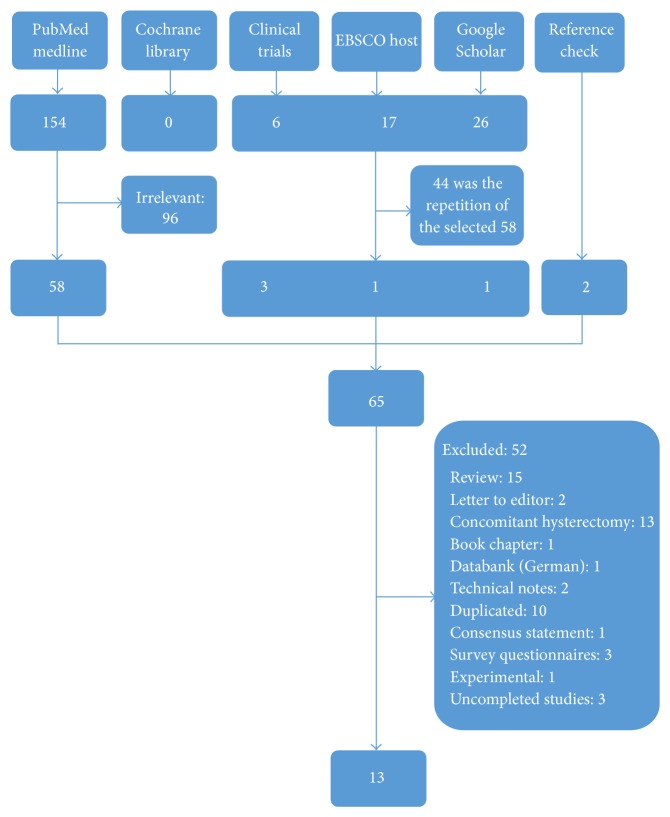
Flowchart of the systematic review.

A total of 154 articles were retrieved from the PubMed/MEDLINE database and no additional study was available in the Cochrane library. After the elimination of the 96 irrelevant studies, the remaining 58 were selected for full-text examination. Google Scholar, EBSCOhost-Academic Search Complete, https://www.clinicaltrials.gov/, and reference cross-checking defined 26, 17, 6, and 2 studies, respectively (Figure 1). After the elimination of repetitive studies in several databases or sources, seven studies were added to the previously selected 58 articles. Studies including inadequate patient data, concomitant hysterectomy, experimental studies, and duplicated data were eliminated and finally 13 articles [3–15] were selected for the analysis (Figure 1). Two studies [10, 11] belonged to the same clinical series and their data were complementary (early and late results). As a result, we analyzed 12 clinical studies contained in 13 articles. Some studies had duplicated results [16–22] and their latest or the most comprehensive versions were accepted for the analysis [6, 10–13].

There were a total of 112 transvaginal appendectomies. Studies originated in Europe, North/South America, and Asia, and two of them included international multicenter data [6, 13]. Publications were generally (11/13) in English but one was in German [9], and one was in Japanese [14]. The patients' ages were generally in the mid-twenties or mid-thirties; however, there was a large age range (18–74 years). Inclusion criteria of the studies for transvaginal appendectomy are summarized in Table 1. All the patients were in the American Society of Anesthesia (ASA) I-II scores. A diagnosis of complicated appendicitis (perforation, abscess, and mass) was usually an exclusion criterion. Morbidly obese patients (body mass index more than 35 kg/m^2^) were also excluded, and the mean body mass index of the patients in this systematic review was 23.2 kg/m^2^ (Table 2) [4, 7–9, 13, 14]. Previous abdominal or pelvic surgery was not an exclusion criterion in all studies and 11% of the patients had a history of abdominal or pelvic surgery [9, 15]. Most of the cases (96%) were of acute appendicitis, while others (4%) were chronic appendicitis or incidental appendectomies.

Mean operating time was 53.3 minutes and ranged from 25 to 130 minutes. There were nine complications reported in 109 cases (8.2%). Four of them were intraoperative complications: appendicular artery hemorrhage (*n*: 3) and inability to sustain the pneumoperitoneum (*n*: 1). Those cases required additional abdominal trocar access and were accepted as a conversion to conventional laparoscopy (3.6%), but no case required conversion to open surgery. Postoperative complications occurred in five patients and all were treated by nonsurgical methods (Table 3). Intra-abdominal abscess, urinary retention, urinary infection, and dyspareunia were treated by percutaneous drainage plus antibiotics, Foley catheter placement, antibiotics, and just waiting, respectively. Mean length of hospital stay was 1.9 days for all studies. The mean length of hospital stay was longer in the German series (3.4 days) because of their national health system, [3, 9], and the mean length of hospital stay was 1.25 days for the rest of the studies.

**Figure 2:**
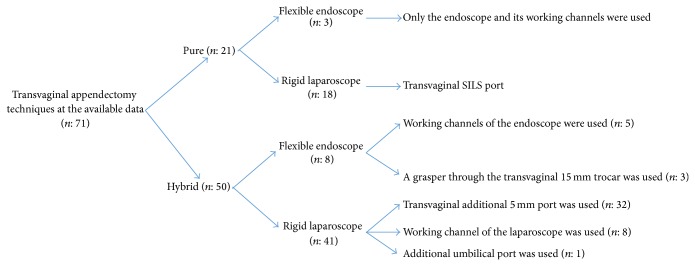
Technical details of the available transvaginal appendectomies.

There was no standard surgical technique for transvaginal appendectomy (Figure 2). Some studies used only the transvaginal access, called a pure or totally transvaginal appendectomy. Others used an abdominal assistance (usually a 5 mm umbilical trocar) to the transvaginal access and are called a hybrid technique (Table 4). Pure transvaginal appendectomy was performed on only 22% of the cases and the remaining were hybrid procedures (78%). When we compared the operating time and the complications for both techniques, there were no differences. The operating times for pure [4, 10] and hybrid [5, 9, 12–15] techniques were 48.3 ± 11.8 minutes and 49.6 ± 25.5 minutes, respectively (*P* = 0.83). Complication rates for pure [3, 4, 10] and hybrid [5, 7–9, 12–15] techniques were 19.0% and 5.5%, respectively (*P* = 0.09). Another technical difference was related to the type of scope. Some authors used flexible endoscopes (20%) and others preferred rigid laparoscopes (80%). Operating times with flexible endoscopes [4, 5, 12, 13] and rigid laparoscopes [9, 10, 13–15] were 71.9 ± 13.3 minutes and 45.2 ± 21.6 minutes, respectively (*P* = 0.0007). Complication rates for using flexible [3–5, 8, 12, 13] and rigid [7, 9, 10, 13–15] scopes were 0% and 11.4%, respectively (*P* = 0.33).

## 4. Discussion

Transvaginal appendectomy during vaginal hysterectomy was first described in 1949 [23] and, at the time, was performed by the gynecologists with the aim of incidental appendectomy [6, 23]. Those studies did not include the acute appendicitis cases and their primary objectives were the treatment of gynecological pathologies, not the appendix. In 2008, the first transvaginal appendectomy without vaginal hysterectomy was reported by Palanivelu and coworkers from India [22] and after a short period of time three more cases were reported one from Germany [3] and two from Georgia [4]. Interestingly, these first three separate reports of transvaginal appendectomies all described the totally transvaginal (pure) technique using only an endoscope [3, 4, 22]. In those cases, there was no abdominal trocar for assistance, nor was there any other transvaginal equipment except endoscopes. All the procedures were performed through the working channels of the endoscopes. Mesoappendixes were transected and the bases of the appendixes were secured with endoloops. Transection of the appendixes was done with scissors [3, 4] or snares [22] through the endoscopic channels. All the specimens were removed with the help of the endoscope and no extraction bags were used. Mean operating time was 95 minutes and ranged between 72 and 135 minutes. Palanivelu et al. reported that, before the first successful case, they experienced three prior conversions to conventional laparoscopic appendectomy due to technical difficulties with this pure endoscopic technique [22]. Bernhardt et al. used the same technique and reported that their case was not an acute appendicitis, but a recurring subacute appendicitis [3]. We can conclude that, despite its minimal invasiveness, technical difficulties limit the application of the pure transvaginal technique to highly selected cases. Technological improvements may make its use more liberal in the future. But for now, this analysis revealed that this earliest described transvaginal appendectomy technique was the least commonly preferred one relative to others subsequently described.

Another technique for pure transvaginal appendectomy was reported using the placement of a single incision laparoscopic surgery (SILS) port into the incised posterior vaginal fornix [10]. There was no abdominal trocar assistance. The authors preferred a 5 mm 30° rigid telescope and two working ports (5 mm and 12 mm) on the SILS port [10]. They divided the mesoappendix with a stapler or an energy device and the appendix was likewise divided with an endoscopic stapler. The specimens were extracted with the endoscopic bags. They reported almost half the operating time (mean 44.4 minutes) of the previously described endoscope-only pure transvaginal appendectomy. Although this new technique seemed more adaptable to daily surgical practice, the authors warned that the SILS port was inadequate as it was too short, which made placement difficult. They concluded that there was still room for innovation in the development of the technique [10].

This analysis pointed out that hybrid procedures with umbilical port assistances were more common (72%) than the pure transvaginal techniques. Hybrid techniques had some advantages over pure ones, such as safer transvaginal introduction under direct vision, transumbilical view when necessary, and two directional working in the abdominal cavity. A hybrid procedure was started with pneumoperitoneum via a Veress needle at the umbilicus and a 5 mm trocar was inserted via the umbilicus to inspect the abdominal cavity. After that a vaginal trocar (10–15 mm) was placed at the posterior fornix of the vagina. An additional working port was created in three different ways in the studies: (i) the channel of the laparoscope was used [7]; (ii) a second 5 mm trocar was inserted from the posterior fornix [15]; or (iii) a second 2.3 mm trocar was placed through the umbilicus [14]. Using a flexible endoscope instead of rigid laparoscope prolonged the operating time. No clear benefits of flexible endoscopes over the rigid scopes were seen in this systematic analysis.

Transferring the surgeons from open surgery to laparoscopic surgery provided the patients with a more comfortable postoperative course and a more rapid recovery. Another promising improvement was the single incision laparoscopic surgery. However, its benefits on postoperative pain and patient recovery were not as amazing as in the previous transfer from open to laparoscopy. A recent meta-analysis found no difference on postoperative pain and length of hospital stay between the single port and the multiport laparoscopic appendectomies [24]. Natural orifice surgery is a novel technique that can have a positive influence on postoperative pain and recovery. An important drawback of this technique may be the unfamiliarity of the access to the abdomen for surgeons who are generally familiar with abdominal incisions or transanal surgeries. A second point that may keep surgeons away from this technique is the idea that complicated surgical equipment is required. This systematic review demonstrated that, from the technical point of view, the equipment required for transvaginal appendectomy was not too distinct from the well-known existing conventional laparoscopic appendectomy equipment. There was no need for special equipment such as long trocars or flexible endoscopes.

Only two studies compared the results of transvaginal and conventional laparoscopic appendectomies [9, 10]. Despite the limited number of the patients in those studies, there was a trend towards shorter hospital stays [9, 10], quicker recovery [10], and less analgesic requirement [10] for the transvaginal groups (Table 5). On the other hand, operating time was longer at transvaginal groups. There was no difference in morbidity. As expected, cosmetic satisfaction was better for the transvaginal group [9]. Recent meta-analyses including thousands of conventional laparoscopic appendectomies demonstrated that the wound infection rate was 3.3–4% and the length of hospital stay was 1.9–2.9 days [24, 25]. When compared to those results, this systematic review demonstrated that transvaginal appendectomy can be a rational alternative to conventional laparoscopic appendectomy in selected patients. It has very low wound infection rates (zero in this study) and short hospital stays (mean 1.9 days).

Today, two matters can limit the widespread use of transvaginal appendectomy. First, there is not enough data of this technique for the morbidly obese patients. There is a considerable amount of obese people in the western countries and transvaginal appendectomy studies are necessary including morbid obese patients. We believe that minimal invasive surgeries like transvaginal appendectomy can have a place for the morbid obese patients in future. Secondly, the risk of delving into the cultural sensitivity of using the vagina as an access point, particularly in third world countries can be a limitation. This can be a problem even in the most promiscuous cultures where virginity still runs some amount of the population.

As a conclusion, appendectomy is one of the most common emergency visceral surgical procedures. The early results of transvaginal appendectomy in this systematic review show some promise for improved postoperative pain and patient recovery. Using hybrid techniques with rigid laparoscopes may provide an easier adaptation for surgeons to this novel appendectomy method. For now, transvaginal appendectomy looks suitable for nonmorbid obese patients (BMI < 35) and noncomplicated appendicitis. Of course, its potential advantages and disadvantages will become clearer in the future with comparative studies. More studies are also necessary on the role of transvaginal appendectomy in some subgroups like morbidly obese patients or perforated appendicitis.

## Figures and Tables

**Table 1:** Inclusion criteria for transvaginal appendectomies.

Parameters	Zorron et al. [6]	Pérez et al. [7]	Roberts et al. [10]	Jacobsen et al. [12]	Noguera et al. [8]	Albrecht et al. [9]	Mofid et al. [15]
Age	NA	18–65	18–65	18–75	18–65	18–78	NA

ASA score	I-II	I-II	I-II	I-II	I-II	NA	NA

Disease severity	Mild symptoms and short duration of disease	No palpable mass No appendicular abscess	Not perforated at clinical and radiological evaluation	<48 hours of the onset No abdominal abscess No abdominal mass No sepsis No diffuse peritonitis	NA	Unclear abdominal discomfort in the sense of appendicitis	NA

Previous surgery	No history of hysterectomy Some centers avoided prior other abdominal surgeries	NA	No abdominal or pelvic surgery	NA	NA	Previous abdominal or pelvic surgery was not a contraindication	Previous abdominal or pelvic surgery was not a contraindication

Virginity or pregnancy	Not virgin Not pregnant	Not virgin Not pregnant	Not pregnant	Not pregnant	NA	NA	NA

BMI	NA	<35	<35	<35	NA	NA	<35

Gynecological pathologies	No vaginal infection No endometriosis No obliterating adhesions in the pouch of Douglas	No gynecological infection	NA	No history of ectopic pregnancy, PID, severe endometriosis, or perineal trauma	NA	NA	No known adhesions in the pelvis

Others	NA	NA	Not retrocecal at preoperative radiological evaluation	No immunosuppressive drug Not immunocompromised No blood thinner medication No platelet inhibitor drugs Normal coagulation tests	Previous delivery of at least one child	NA	No malignancy No previous pelvic radiotherapy

NA: not available, BMI: body mass index, ASA: American Society of Anesthesiology, and PID: pelvic inflammatory disease.

**Table 2:** Patient and article details.

Author	Year	Country	Number	Age (mean)	Appendectomy indication	BMI	Prior surgery
Bernhardt et al. [3]	2008	Germany	1	28	Subacute	NA	No
Tabutsadze and Kipshidze [4]	2009	Georgia	2	22, 28	Acute	22.2 & 23.5	No
Shin et al. [5]	2010	Korea	1	74	Acute	Not obese	No
Zorron et al. [6]	2010	International	37	NA	Acute	NA	Some
Pérez et al. [7]	2011	Cuba	8	29.6 (18–42)	Acute	<35	NA
Noguera et al. [8]	2011	Spain	4	NA	Acute (2), incidental (2)	NA	NA
Roberts et al. [10]	2012	USA	18	31.3	Acute	23.7	No
Albrecht et al. [9]	2013	Germany	30	33.9	Acute	23.7	Yes (5)
Jacobsen et al. [12]	2014	USA	3	NA	Acute	NA	NA
Arezzo et al. [13]	2013	International	5	31.2 (23–42)	Acute	18.9 (18-19)	NA
Wada et al. [14]	2013	Japan	1	50	Acute	24.2	No
Mofid et al. [15]	2013	Germany	2	NA	Chronic	NA	Yes (1)

BMI: body mass index.

**Table 3:** Results of transvaginal appendectomies.

Author	Number	Operating time (min) mean (range)	Complications (number)	Conversion to laparoscopy (number)	Length of stay (mean and range)
Bernhardt et al. [3]	1	NA	No	No	3
Tabutsadze and Kipshidze [4]	2	76 & 88	No	No	1.25 & 1.5
Shin et al. [5]	1	60	No	No	3
Zorron et al. [6]	37	60.5 (90 for flexible)	Appendicular artery hemorrhage (3)	Appendicular artery hemorrhage (3)	1.3
Pérez et al. [7]	8	48.3 (37–75)	No	No	1.1 (<1-2)
Noguera et al. [8]	4	61	No	No	NA
Roberts et al. [10]	18	44.4	Intra-abdominal abscess (1) Urinary retention (1) Unable to sustain pneumoperitoneum (1)	Unable to sustain pneumoperitoneum (1)	1.1
Albrecht et al. [9]	30	44.3	Urinary infection (1) Dyspareunia (2)	No	3.4
Jacobsen et al. [12]	3	71 (55–80)	NA	No	NA
Arezzo et al. [13]	5	Rigid: 42.5 (40–45) Flexible: 70 (60–90)	No	No	Rigid: 1.5 (1-2) Flexible: 1.3 (1-2)
Wada et al. [14]	1	130	No	No	1
Mofid et al. [15]	2	25 & 32	No	No	NA

**Table 4:** Technical details of transvaginal appendectomies.

Author	Number	Vaginal trocar	Umbilical assistance	Working access	Flexible or rigid scope
Bernhardt et al. [3]	1	No	No (pure)	Endoscope channel	Flexible
Tabutsadze and Kipshidze [4]	2	NA	No (pure)	Endoscope channel	Flexible
Shin et al. [5]	1	15 mm	5 mm (hybrid)	Endoscope channel	Flexible
Zorron et al. [6]	37	10 or 12 mm	No (pure) or 5 mm (hybrid)	NA	Both
Pérez et al. [7]	8	11 mm	5 mm (hybrid)	Laparoscope channel	Rigid
Noguera et al. [8]	4	15 mm	5 mm (hybrid)	Endoscope channel	Flexible
Roberts et al. [10]	18	SILS port	No (pure)	TV SILS port	Rigid
Albrecht et al. [9]	30	12 mm	5 mm (hybrid)	TV 5 mm port	Rigid
Jacobsen et al. [12]	3	15 mm	5 mm (hybrid)	Through TV 15 mm trocar	Flexible
Arezzo et al. [13]	5	12 mm	5 mm (hybrid)	NA	Flexible (3), rigid (2)
Wada et al. [14]	1	12 mm	5 mm (hybrid)	2.3 mm umbilical trocar	Rigid
Mofid et al. [15]	2	5 and 10 mm	5 mm (hybrid)	TV 5 mm trocar	Rigid

TV: transvaginal.

**Table 5:** Comparison of transvaginal and conventional laparoscopic appendectomies.

Parameters	Studies	Transvaginal	Conventional	*P*
Operating time (minutes)	Albrecht et al. [9] (*n*: 30 versus *n*: 30)	44.3 ± 22.1	33.5 ± 10.0	0.02
	Roberts et al. [10] (*n*: 18 versus *n*: 22)	44.4 ± 4.5	39.8 ± 2.6	<0.01

Hospital stay (days)	Albrecht et al. [9] (*n*: 30 versus *n*: 30)	3.4 ± 1.2	5.0 ± 2.7	<0.01
	Roberts et al. [10] (*n*: 18 versus *n*: 22)	1.1 ± 0.1	1.2 ± 0.1	<0.01

Complications	Albrecht et al. [9] (*n*: 30 versus *n*: 30)	Urinary tract infection (1)	No	1.00
	Roberts et al. [10] (*n*: 18 versus *n*: 22)	Intra-abdominal abscess (1) Urinary retention (1)	Intestinal obstruction (1) Urinary retention (1)	1.00

Opioid requirement (mg)	Albrecht et al. [9] (*n*: 9 versus *n*: 9)	12.8 ± 7.0	14.7 ± 5.2	0.52
	Roberts et al. [10] (*n*: 18 versus *n*: 22)	8.7 ± 2.1	23.0 ± 3.4	<0.01

Return to normal activity after 2 weeks	Albrecht et al. [9] (*n*: 30 versus *n*: 30)	70%	59%	0.58

Return to normal activity (days)	Roberts et al. [10] (*n*: 18 versus *n*: 22)	3.3 ± 0.4	9.7 ± 1.6	<0.01

Return to work (days)	Roberts et al. [10] (*n*: 18 versus *n*: 22)	5.4 ± 1.1	10.7 ± 1.5	<0.01

Cosmetic satisfaction	Albrecht et al. [9] (*n*: 30 versus *n*: 30)	100%	80%	0.02
	Roberts et al. [10] (*n*: 18 versus *n*: 22)	NA	NA	NA

NA: not available.
