# Corrigendum to “Transvaginal Appendectomy: A Systematic Review”

**DOI:** 10.1155/2015/527140

**Published:** 2015-06-28

**Authors:** Cuneyt Kayaalp, Kerem Tolan, Mehmet Ali Yagci

**Affiliations:** Department of Surgery, Turgut Ozal Medical Center, Inonu University, 44315 Malatya, Turkey

Previously our systemic review about the transvaginal appendectomies was published in this journal [1]. As we mentioned in our review, “transvaginal appendectomy during vaginal hysterectomy was first described in 1949 and, at that time, it was performed by the gynecologists with the aim of incidental appendectomy. Those studies did not include the acute appendicitis cases and their primary objective was the treatment of gynecological pathology, not the appendix.” After that we continued as follows: “in 2008, the first transvaginal appendectomy without vaginal hysterectomy was reported by Palanivelu and coworkers from India and after a short period of time three more cases were reported one from Germany and two from Georgia.”

After the publication of our study, we received an email from Dr. Daniel Alberto Tsin from Mount Sinai Hospital of Queens Long Island City (NY), Department of Gynecology (24 May 2015). He pointed out that they had also a small number of published series (three cases) about transvaginal appendectomy in 2001 [2]. We sadly realized that we failed to notice their work, due to the gynecological origin of their study. When we take in consideration his work, we may say that first transvaginal appendectomy (without hysterectomy) was in fact performed by Dr. Tsin in 2001 and coworkers [2]. But we must also mention that, in all these three cases, a hybrid technique (10–12 mm transvaginal trocar with accompanying two 3 and 5 mm abdominal trocars) was used and none of these three cases were acute appendicitis. Lastly, NOTES (Natural Orifice Transluminal Endoscopic Surgery) as an acrimonious was not present at that time; thus culdolaparoscopy was the name of their technique.
